# Annotations

**Published:** 1891-07-04

**Authors:** 


### Annotations.

**Doctors' Fees.**

"A doctor who works for nothing is good for nothing" says an old Jewish proverb. Two or three sorts of people might reflect upon this proverb with advantage: firstly, certain members of the race which originated it; secondly, doctors themselves who cut down their fees to a minimum; and, thirdly, patients who love and insist upon cheap doctors. There are no very obvious criteria which enable us to say with certainty exactly what a doctor's fee ought to be. Some persons think that the family physician should never charge more than a guinea a visit, or less than half-a-crown, to persons who live within a mile of his own house. How far this is sound wisdom need not here be discussed: but there are certain considerations which will help both doctors and patients to apply to their own cases the grand rule of doing as they would be done by. A doctor, it is manifest, is an educated man. If, therefore, he be a fairly competent practitioner, he should be able to earn the income of an educated man. That is to say, doctors' fees should be so regulated that a good practitioner, who is fully employed for ten or twelve hours a-day, should be able to secure such a measure of remuneration as will house, feed, and clothe himself and his family, and also educate the latter, in the way in which other educated people are usually housed, fed, clothed, and educated. This is but justice. There is a minimum below which even political economy does not allow the worker to fall. It claims for the poorest labourer at least the means of subsistence. By parity of reasoning, the educated doctor may claim, even by the laws of political economy, the means of educated subsistence. But there is a second consideration, which also ought to be of great weight both with patients and doctors. The medical practitioner is a brain worker. If any body smiles at this, thinking his own doctor to be somewhat poor in brains, he should ask himself whether it be not time to try a new one. The doctor is emphatically a brain worker if he practice his business honestly. But men who work with their brains should not be worried. A worried brain is a weakened brain. Nothing worries the brain more than a life-long struggle to make both ends meet. Every patient who pays his doctor three and sixpence a visit, when he ought to pay him five shillings, is not only deducting a third from his fee, but a third from his brain power. Nay, he is possibly even deducting a third from his conscience-power also. He is thereby not only exposing himself to the risk of having the illnesses of his family unduly prolonged, but also to the possibility of having the full amount and more than the full amount extorted from him by other means. For the doctor whose brains and conscience are impaired by unjust usage has questionable methods of his own for securing what he considers his due. Full pay for full work the physician should demand as his right. His acts of pure charity, which of necessity are many, come under a different rule. The urgent competition among medical men tends naturally to the lowering of their fees; but if the public is wise it will, for its own sake, do nothing to aid in the degradation of the medical profession. Every sensible head of a household will show his good sense by choosing for his family doctor a man who has the manliness and self-respect to set a reasonable value upon his own services. It is a very sound rule, that he who insists upon being paid for his work generally takes care to do work which is worth paying for.

**Wanted, an English Haussmann.**

A reviewer of the *Memoirs of Baron Haussmann* concludes with the weighty summing-up that "he lived honest and died poor." It might have been added that he made a new Paris and a capital for the world. It is not once in a century that a country produces such a man as Baron Haussmann: it is not once in ten centuries that the contemporaries of such a man have the sense to recognise his worth and to give him his way. Fortunately for Paris, and for France and the world, the Emperor saw in Haussmann the power and the sagacity that were needed, and he had the independence of mind to appoint him to the practical and permanent headship of Paris municipal life. Merely to recount what Haussmann did during his tenure of office would be to more than fill the space at our disposal. It is not necessary, however, since most persons who are interested in the large questions of public health, and municipal beauty and utility, have made themselves more or less familiar with his work. What we desire to do is to affirm the desirableness of such a man as Haussmann for London, and for every other large centre of population in the United Kingdom. The magnitude of London is alarming, and it is rapidly becoming dangerously unmanageable. Perfect sanitation is indispensable, and it is possible. But it is only possible if the sanitation is carried out under one single system, and by one single authority. What is wanted, if it could be got, is one capable head, and one free hand. The atmosphere, the water supply, and the drainage of London are the rocks on which its health and activity are for ever threatening to split and go to pieces. But, in addition to these, there are the questions of easy transit through the streets, and of municipal beauty and dignity. London never has been managed in such a way as to conserve all the vital interests here mentioned. Even now the County Council proceeds with halting steps, and with the eternal fear of the taxpayer paralysing its soul. A Haussmann is wanted: a man who would be of no party; a man who would not fear the taxpayer and his vote; a man whose appointment should be for life or fault. Such a man would have to choose between serving the best permanent interests of the people, and merely pleasing the people's fancy. If he had the courage and the clearsightedness to persistently do the former, the English people would first metaphorically tear him to pieces in stupid tax-hating rage, and afterwards they would raise him to the highest possible pinnacle of fame and honour. They would insist that he should end his days in the peerage, and be buried in Westminster Abbey. Socialism is the parrot-cry of the moment. It is not socialism that is needed in our complex civilisation, but individualism: every strenuous person to do the most strenuous that is in him to the last day of his life. Less than this means rottenness and decay within a measurable time. A Haussmann would not only give us healthy and beautiful cities, but an unflinching example of steady self-reliance which would put new and irresistible life into our sickly individualism.

**The Manchester Misadventures.**

Two cases of deaths after the administration of chloroform were investigated last week by the Deputy Coroner for Manchester, Mr. S. Smelt. In the course of the enquiry the coroner stated that since January of the present year five deaths had taken place, under circumstances similar to those of the two he was then investigating. He also affirmed that during the last five years the number of deaths following upon the administration of chloroform at Manchester had not been so great as during the last five months. Mr. Thompson, one of the house surgeons of the Infirmary, gave it as his opinion that death in one of the cases was due to syncope, produced by the chloroform. In the other case, which was that of a railway guard, certain circumstances rendered it at least doubtful whether the chloroform was the sole cause or, indeed, in any sense a determining factor in the unhappy result. The chloroform had been supplied by Messrs. Woolley, of Manchester, and Mr. H. Woolley, representing the firm, gave evidence. He stated that it was manufactured differently from ordinary chloroform, and was somewhat cheaper in price. We have since learnt from Mr. Woolley himself that the chloroform supplied by his firm is extensively used in America, and has been supplied to a considerable number of private practitioners in this country, and also to one of the largest London general hospitals. There has been no case of death reported from any of these sources. The chloroform in question is, in America, held to be equal to the best. Our object in writing is to try to prevent a public scare among hospital patients and their friends. It is certainly unusual that five deaths should occur at one institution within such a short period. But of the two investigated last week it is obvious that one at least was probably not due to the chloroform at all; and that there was nothing whatever to show conclusively that the other was due to the chloroform alone. It is quite possible that the same results might appear if the cause of death in the other three instances were scientifically investigated. The whole series of misfortunes may be nothing more than a series of coincidences. We point this out as a matter of scientific interest. If this particular chloroform is to be banned on the strength of such a partial and comparatively unscientific enquiry as has been carried out, we need not expect any new experiments with anæsthetics of this kind for the next twenty years. There is eminently a case for scientific investigation; but there is as yet no proof of definite chloroform poisoning, and no reason therefore for wide-spread and premature alarm.

